# Factors Shaping Perceptions of AI Tools Among a Nationally Representative Sample of US Adults

**DOI:** 10.1093/geroni/igaf122.1552

**Published:** 2025-12-31

**Authors:** Benjamin Katz, Norhan Abdelgawad, Daniel Friedberg, Patrick Roberts, Shalini Misra

**Affiliations:** Virginia Tech, Blacksburg, Virginia, United States; Virginia Tech, Arlington, Virginia, United States; Virginia Tech, Arlington, Virginia, United States; Virginia Tech, Arlington, Virginia, United States; Virginia Tech, Arlington, Virginia, United States

## Abstract

Individuals throughout the life span are increasingly faced with challenging decisions regarding the adoption of generative AI tools in a variety of workplace contexts. In this nationally representative study of N = 500 US adults collected via the Prolific platform, we examined how a variety of demographic factors, thinking dispositions, and industry-types, including both K-12 and higher education (N = 37), influenced how individuals considered risk and utility of generative AI tools in their work. AI-relevant scales included the General Attitudes towards AI scale, an AI risk and benefits scale, AI frequency and expertise, and a scale for the assessment of non-experts’ AI literacy. While higher education and K-12 industry status was not linked to differences in differential AI perceptions, age was closely linked to a variety of outcomes, including perceived benefits of AI, perceived risk of AI, and the optimal role of AI in workplace applications. For example, older individuals were in some cases more likely to agree strongly with statements emphasizing the potential benefits of AI and were somewhat less likely to agree with statements emphasizing the risks of AI in workplace contexts. Further analyses identified nuanced links with thinking dispositions including one’s likelihood to engage in cognitively challenging activities and how susceptible one was to everyday cognitive failures. These findings may have implications for future curricula and programming designed to help individuals throughout the life span manage the proliferation of generative AI tools in workplace contexts, including within gerontology education.

